# Dextran Fluorescent Probes Containing Sulfadiazine and Rhodamine B Groups

**DOI:** 10.3390/molecules27196747

**Published:** 2022-10-10

**Authors:** Bi-Jie Bie, Xiao-Rui Zhao, Jia-Rui Yan, Xi-Jun Ke, Fan Liu, Guo-Ping Yan

**Affiliations:** 1 School of Materials Science and Engineering, Wuhan Institute of Technology, Wuhan 430205, China; 2 Faculty of Science, University of Melbourne, Grattan Street, Parkville, VIC 3010, Australia

**Keywords:** fluorescent imaging, fluorescent probe, dextran, rhodamine B, sulfadiazine

## Abstract

Fluorescent imaging has been expanded, as a non-invasive diagnostic modality for cancers, in recent years. Fluorescent probes in the near-infrared window can provide high sensitivity, resolution, and signal-to-noise ratio, without the use of ionizing radiation. Some fluorescent compounds with low molecular weight, such as rhodamine B (RhB) and indocyanine green (ICG), have been used in fluorescent imaging to improve imaging contrast and sensitivity; however, since these probes are excreted from the body quickly, they possess significant restrictions for imaging. To find a potential solution to this, this work investigated the synthesis and properties of novel macromolecular fluorescent compounds. Herein, water-soluble dextran fluorescent compounds (SD-Dextran-RhB) were prepared by the attachment of RhB and sulfadiazine (SD) derivatives to dextran carrier. These fluorescent compounds were then characterized through IR, ^1^H NMR, ^1^^3^C NMR, UV, GPC, and other methods. Assays of their cellular uptake and cell cytotoxicity and fluorescent imaging were also performed. Through this study, it was found that SD-Dextran-RhB is sensitive to acidic conditions and possesses low cell cytotoxicities compared to normal 293 cells and HepG2 and HeLa tumor cells. Moreover, SD-Dextran-RhB demonstrated good fluorescent imaging in HepG2 and HeLa cells. Therefore, SD-Dextran-RhB is suitable to be potentially applied as a probe in the fluorescent imaging of tumors.

## 1. Introduction

In recent years, fluorescent imaging (FI) has been expanded as a non-invasive invasive imaging technique in preclinical and clinical applications [1,2]. Moreover, FI also is widely used in molecular bioimaging and the diagnosis of cancers, as the near-infrared (NIR) fluorescent probes provide high sensitivity, resolution, and signal-to-noise ratio, without the use of ionizing radiation [3,4,5,6].

Currently, some compounds are synthesized and evaluated as potential fluorescent probes for labeling and detecting tumor cells and tissues in FI, to improve imaging contrast and sensitivity [7,8]. However, the widely used fluorescent probes with low molecular weight, such as rhodamine B (RhB) and indocyanine green (ICG), have been used in FI to improve imaging contrast and sensitivity. However, since these probes are excreted from the body quickly and usually demonstrate no tumor-targeting biodistribution, they then possess significant restrictions for the imaging of tumors [9,10,11]. To find a potential solution to this, a tumor-targeting fluorescent probe is an ideal trend to achieve bioimaging with high sensitivity, resolution, and imaging-contrast enhancement, as well as suitable long duration and excretion time in vivo [12,13,14,15,16,17,18].

Antibody-labeled quantum dots (QDs) have been recently receiving widespread interest as a fluorescent probe in cell imaging, biological tracking, and molecular detection. This nanomaterial can provide tumor-targeting fluorescent imaging based on the specific affinity reaction between antibody and antigen. However, there are some fundamental drawbacks, such as the high toxicity of QDs and high price of the antibody, which hamper their wide application [19,20]. Due to this, one approach to water-soluble macromolecular fluorescent probes has also attracted widespread attention in the field of FI, in recent years. Examples include water-soluble polyaspartamide and fullerenol fluorescent probes, which were reported to have low cell cytotoxicity, high uptake, and good FI in HeLa tumors [21,22].

Dextran is a water-soluble polysaccharide that possesses low immunogenicity, good biocompatibility, biodegradability, medicine permeability, and ease of chemical modification. So, it is used as a biodegradable polymeric carrier in biomedical applications [23,24]. In previous works, dextran-containing gadolinium complexes were shown to have a greatly enhanced magnetic resonance imaging (MRI) contrast between normal and diseased popliteal lymph nodes, while providing a prolonged duration of stay in the lymphatic system in rabbits [25,26,27].

Sulfadiazine (SD) has been applied as a tumor-targeting group in bioimaging and drug-delivery systems, due to its highly specific uptake by the Walker carcinoma and Yoshida sarcoma [28,29,30]. Some SD-modified small molecular and polymeric gadolinium complexes were reported as tumor-targeting MRI contrast agents, which greatly enhanced the contrast of MR images in Ehrlich ascites carcinoma and hepatoma in mice. However, the tumor-targeting mechanism of SD derivatives has not been clearly revealed, so a lot of in-depth research is still needed. One of the more recognized mechanisms proposed is similar to the tumor-targeting mechanism of folic acid, due to the structures of SD being similar to those of p-aminobenzoic acid, a raw material in the synthesis of folic acid in tumors. On the surface of the cell membrane of some tumors, a special protein with the ability to specifically recognize and bind with folic acid, referred to as the folic acid receptor (FR), is overexpressed, which allows the drug molecules combined with SD to be introduced into these tumor cells through the special action mentioned above [31,32,33].

**Scheme 1:**
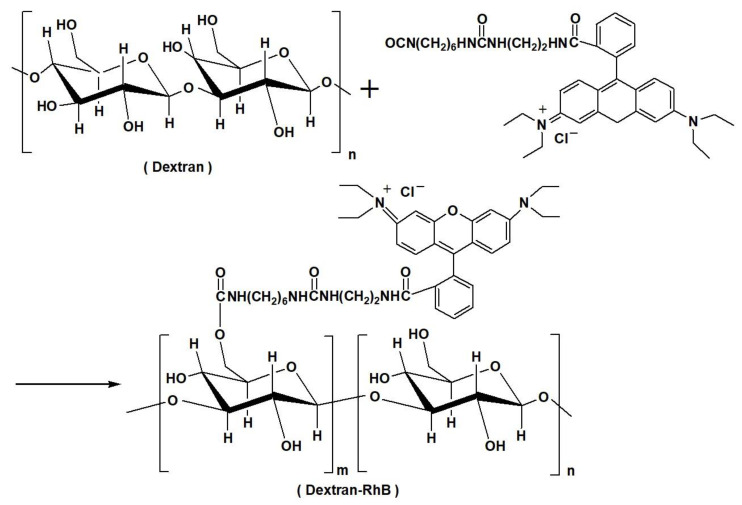
Synthetic route to dextran containing rhodamine B (Dextran-RhB).

Herein, macromolecular fluorescent compounds (SD-Dextran-RhB) were prepared by the attachment of both SD and RhB derivatives [34,35,36] to dextran, as a polymer carrier (Scheme 1). In the design of the SD-Dextran-RhB molecular structure, RhB was chosen as a fluorescent group, and SD was expected to apply its tumor-targeting function. The products were further characterized by ^1^H NMR, IR, ultraviolet–visible absorption spectroscopy (UV), gel permeation chromatography (GPC), etc., and then evaluated as potential macromolecular fluorescent probes in the FI of tumors.

## 2. Materials and Methods

### 2.1. Materials

Sulfadiazine sodium (SDNa) and rhodamine B derivative modified with diisocyanate (RhB-NCO) were prepared, in accordance with the procedures cited in the references [37,38]. The liver hepatocellular cells (HepG2), HeLa cells, and normal cells (human embryonic kidney cell 293 cells) were supplied by the China Center for Type Culture Collection of Wuhan University, China, and raised in accordance with the process cited in the references [39].

### 2.2. Synthesis of Dextran Containing Rhodamine B (Dextran-RhB)

A solution of rhodamine B derivative modified with diisocyanate (RhB-NCO, 3.36 g, 5 mmol) in 20 mL of DMSO was added slowly to a solution of dextran (2.78 g, 0.017 mol of repeat structural unit) and dibutyl tin dilaurate (DBTL, 3.06 mL, 3.25 g, 0.017 mol) in dimethyl sulfoxide (DMSO, 20 mL), in a nitrogen atmosphere at 20 °C. The reaction mixture was stirred for 24 h at 20 °C, filtered, and precipitated against ethanol. The precipitate was reprecipitated from DMSO against ethanol, filtered, and dried in vacuo. After dialysis using distilled water for 48 h, the solution was distilled to remove water, and the solid was dried in vacuo to make a pink RhB-modified dextran (Dextran-RhB, 3.1 g, 49%) (Scheme 1). ^1^H NMR (DMSO-d_6_, ppm) δ: 7.77 (s, 1H, C_6_H_3_), 7.53–7.44 (m, 2H, C_6_H_3_), 6.98 (s, 1H, C_6_H_4_), 6.35 (d, *J* = 6.7 Hz, 6H, C_6_H_4_), 4.90 (dd, *J* = 24.7, 4.7 Hz, 92H, OCH_2_O), 4.72–4.62 (m, 40H, OCH), 4.52 (d, *J* = 5.8 Hz, 38H, OCH), 3.75 (s, 43H, OCH), 3.63 (s, 39H, OCH), 3.21 (s, 80H, OCH_2_), 1.23 (s, 16H, CH_3_), 1.08 (s, 12H, CH_3_); ^13^C NMR (DMSO-d_6_, ppm) δ: 153.00, 148.86, 128.68, 108.65, 105.35, 44.13, 40.92, 26.60, 12.90 (RhB), 98.71, 97.78 (dextran); IR (KBr, cm^−1^) ν_max_: 3450 (OH), 2920 (C-H), 1650, 1470 (COO, CONH), 1384, 1270 (C-N), 1160, 1010 (C-O), 947, 912, 866, 765, 618 (C_6_H_4_).

### 2.3. Synthesis of Bromine-Substituted Dextran Containing Rhodamine B (Br-Dextran-RhB)

**Scheme 2:**
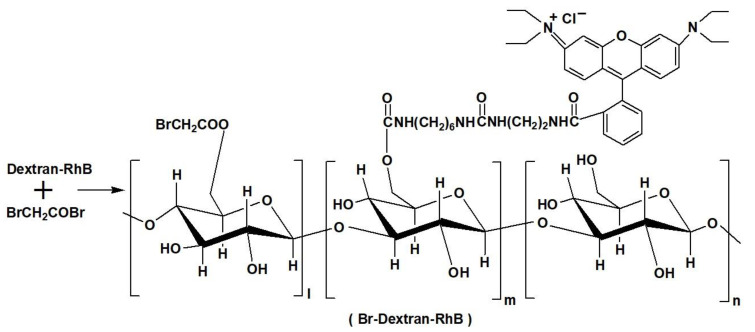
Synthetic route to bromine-substituted dextran containing rhodamine B (Br-Dextran-RhB).

Dextran-RhB (3.0 g, 0.01 mol of repeat structural unit) was dissolved in DMSO (30 mL), and then bromoacetyl bromide (0.18 mL, 2 mmol, 20% molar ratio of repeat units of dextran) was added. Pyridine (0.79 g, 0.01 mol) was added slowly to the mixture solution at 0 °C. The reaction mixture was stirred for 72 h at 20 °C, then filtered, and precipitated against ethanol. The solid was collected and dried in vacuo to obtain bromine-substituted dextran containing rhodamine B (Br-Dextran-RhB, 3.1 g, 80%) (Scheme 2).

### 2.4. Synthesis of Dextran Fluorescent Compounds (SD-Dextran-RhB)

**Scheme 3:**
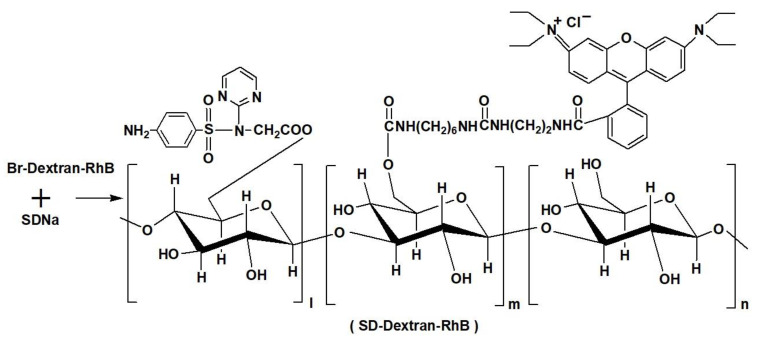
Synthetic route to dextran fluorescent compounds (SD-Dextran-RhB).

A solution of Br-Dextran-RhB (2.5 g) in DMSO (30 mL) was added to a solution of sulfadiazine sodium (SDNa, 0.456 g, 1.6 mmol) in DMSO (20 mL). Subsequently, 10% tetrabutylammonium hydroxide solution (2.0 mL, 8.4 mmol) was added. The mixture was stirred for 72 h at 20 °C. The reaction mixture was filtered and precipitated against ethanol (300 mL). The precipitate was dialyzed using distilled water for 48 h, then the solution was distilled to remove water, and the solid was dried in vacuo to make a pink dextran fluorescent compound containing RhB and SD groups (SD-Dextran-RhB, 2.37 g, 85.2%) (Scheme 3).

SD-Dextran-RhB: ^1^H NMR (DMSO-d_6_, ppm) δ: 8.14 (s, 1H, C_4_H_4_N_2_), 7.90–7.77 (m, 1H, C_4_H_4_N_2_), 7.49–7.45 (s, 4H, C_6_H_3_), 6.99 (d, *J* = 12.7 Hz, 2H, C_6_H_4_), 6.51–6.26 (m, 10H, C_6_H_4_), 4.92 (d, *J* = 20.8 Hz, 199H, OCH_2_O), 4.67 (s, 91H, OCH), 4.55 (s, 77H, OCH), 3.73 (s, 92H, OCH), 3.63 (s, 104H, OCH), 3.21 (s, 167H, OCH_2_), 1.07 (s, 15H, CH_3_); ^13^C NMR (DMSO-d_6_, ppm) δ: 157.74, 152.99, 148.86, 130.66, 129.58, 129.16, 128.66, 112.38, 108.65, 108.59, 98.68, 97.76, 73.81, 72.89, 72.48, 72.31, 70.85, 70.61, 66.57, 44.13, 26.60, 12.90; IR (KBr, cm^−1^) ν_max_: 3440 (OH), 2920 (C-H), 1650, 1520, 1460 (COO, CONH), 1400, 1380 (C-N), 1270 (SO), 1160, 1110, 1040, 1010 (C-O), 946, 912, 868, 762, 668 (C_6_H_4_); UV-Vis (H_2_O, nm) λ_max_: 254, 565. The average grafted mole ratio of attached SD and RhB to dextran repeat units (mol%) was 1% and 2.9% (as determined by ^1^H NMR), respectively.

### 2.5. Cell Cytotoxicity Test

HepG2, HeLa, and normal 293 cells (2 × 10^5^/mL of density) were plated in 96-well plates in RPMI-1640 media (100 µg/mL streptomycin, 100 units/mL penicillium, 10% fetal bovine serum, 2 × 10^4^ cells/well). After incubation for 24 h in an incubator (37 °C, 5% CO_2_), growth medium was separated, and 200 µL of SD-Dextran-RhB solution (0.025, 0.05, 0.1, 0.25 or 0.5 mg/mL) in growth medium was added. After incubating for 48 h, a thiazolyl blue (3-[4,5-dimethylthiazol-2-yl]-2,5-diphenyltetrazolium bromide (MTT, 5 mg/mL, 20 µL) solution in the phosphate buffer saline solution (PBS) was added in each well. The cells were cultured for 4 h, growth medium was removed again, and then DMSO (200 µL) was added. The cells were shaken for 30 min at 20 °C. The absorbance (optical density: OD_490_) was investigated at 490 nm using a DG-3022A ELISA-Reader (Hercules, CA, USA) and analyzed.

### 2.6. Cell Uptake Assay

HepG2 cells (5 × 10^5^/mL) were implanted in 24-well plates in DMEM media (100 µg/mL streptomycin, 10% fetal bovine serum and 100 units/mL penicillium). After incubation for 48 h in an incubator (37 °C, 5% CO_2_), growth medium was separated, and the solution of SD-Dextran-RhB (200 µL, 0.25 mg/mL) and Dextran-RhB (200 µL, 0.25 mg/mL) in growth medium or pure growth medium were added, respectively. After incubation for 2 h, growth medium was separated again, and cells were rinsed with PBS three times. Subsequently, cells were fixed with paraformaldehyde (0.5 mL, 4%) for 10 min and stained with 4’,6-diamidino-2-phenylindole (DAPI, 5 μg/mL) for another 10 min. The cell morphology and density were observed by Leica confocal laser scanning microscope (CLSM) (TCS SP8, Heidelberg, Germany).

### 2.7. Fluorescent Imaging Assay

HeLa cells (5 × 10^5^/mL) were implanted in 24-well plates in RPMI-1640 media (100 µg/mL streptomycin, 10% fetal bovine serum and 100 units/mL penicillium) and cultured for 48 h in an incubator (37 °C, 5% CO_2_). Then, growth medium was separated, and 200 µL of SD-Dextran-RhB solution (0.25 mg/mL) in growth medium or pure growth medium was added. After incubation for 2 h, growth medium was then separated again, and cells were rinsed with PBS three times. Subsequently, cells were fixed using paraformaldehyde (4%, 0.5 mL) for 10 min. The cell morphology and density were evaluated using an IX-70 inverted fluorescence inverted microscope.

## 3. Results and Discussion

### 3.1. Synthesis and Characterization

**Figure 1:**
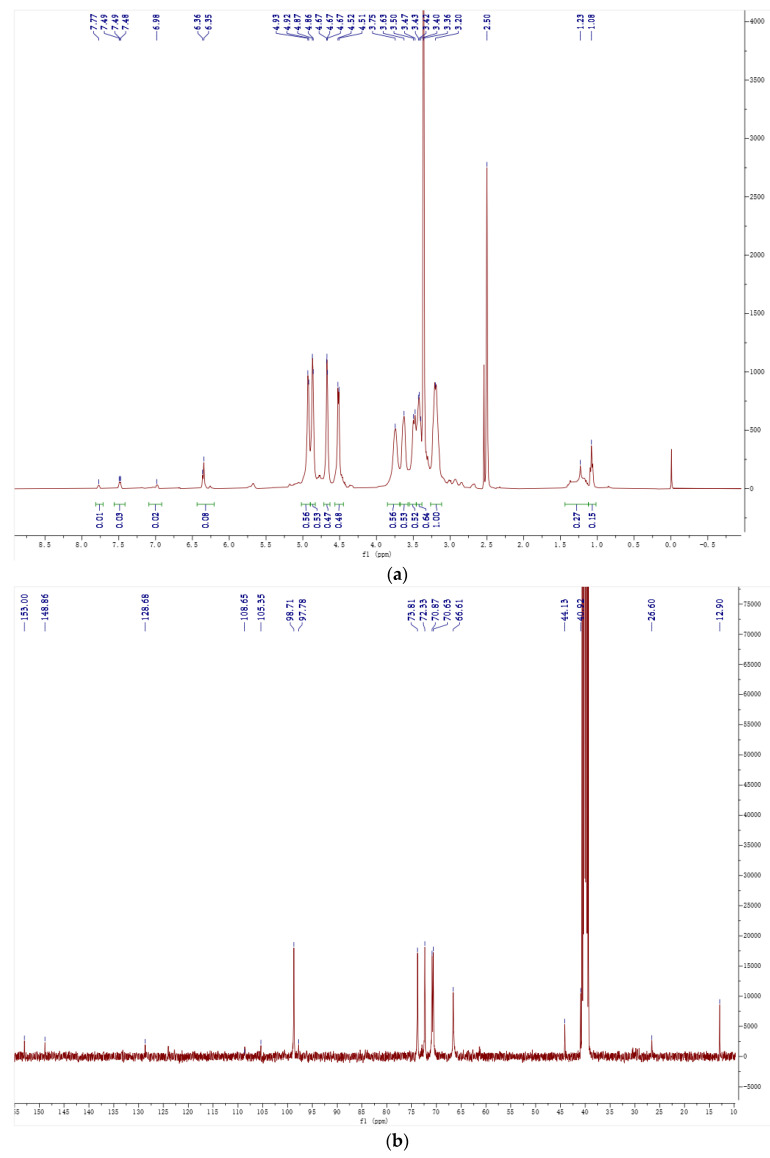

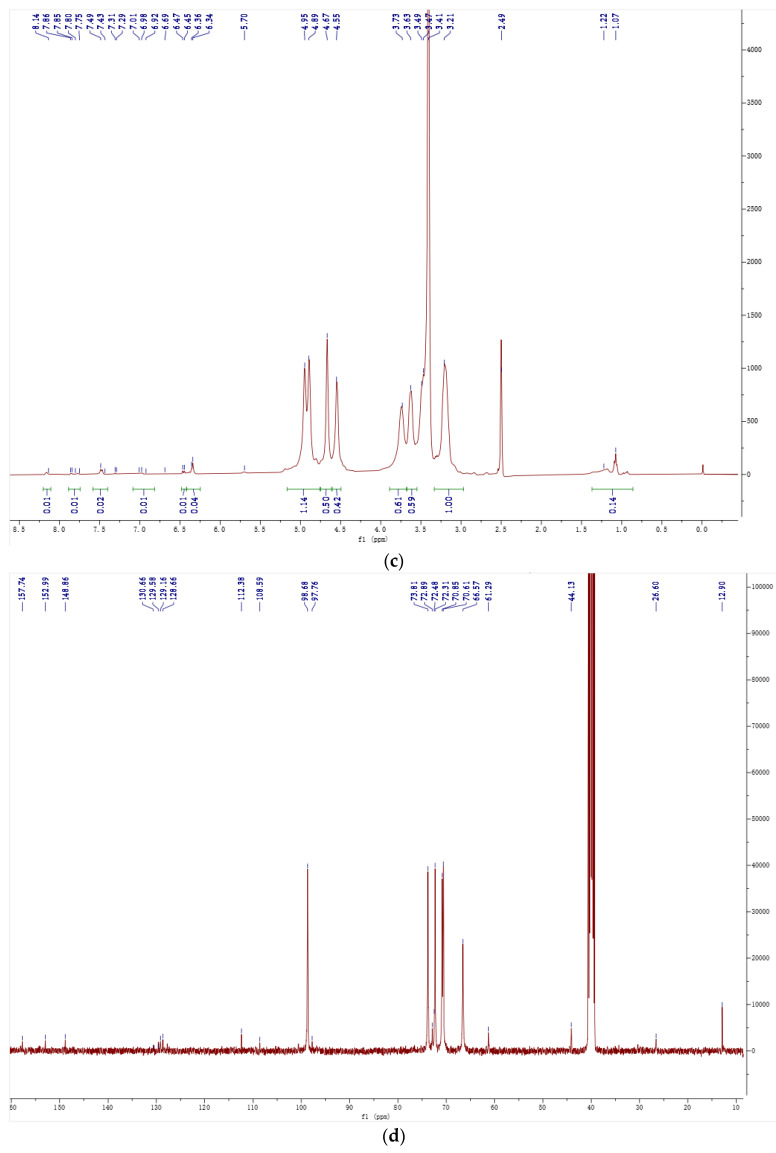
^1^H NMR and ^1^^3^C NMR spectra of Dextran-RhB (**a**,**b**) and SD-Dextran-RhB (**c**,**d**).

**Figure 2:**
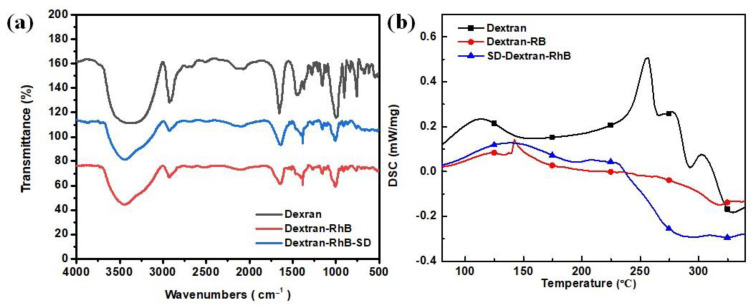
IR spectra (**a**) and DSC curves (**b**) of SD-Dextran-RhB.

**Figure 3:**
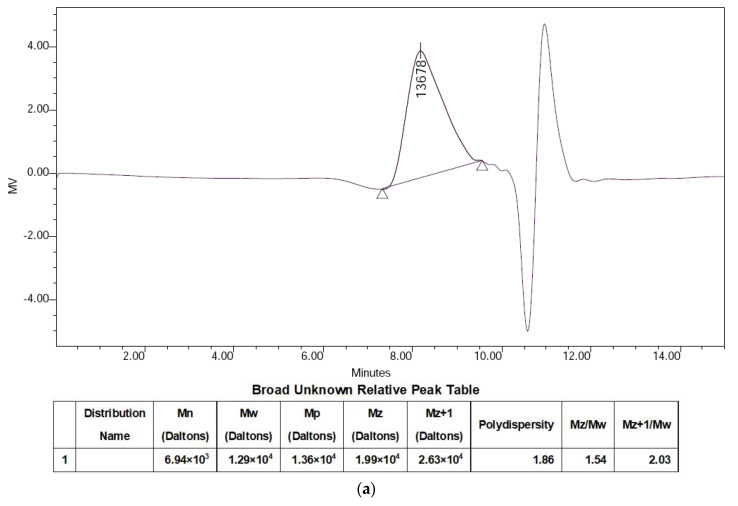

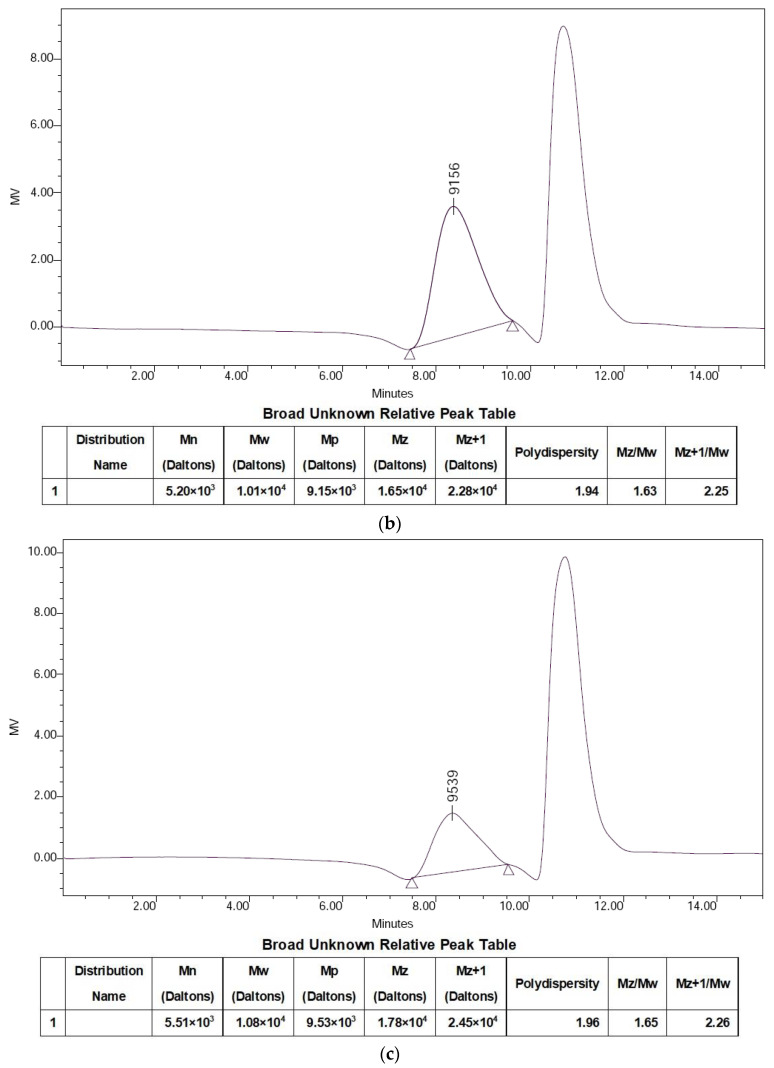
GPC of dextran (**a**), Dextran-RhB (**b**), and SD-Dextran-RhB (**c**).

Both RhB as a fluorescent group and SD as a tumor-targeting group were incorporated to dextran to synthesize dextran fluorescent probes (SD-Dextran-RhB) (Scheme 1, Scheme 2 and Scheme 3). Dextran-RhB and SD-Dextran-RhB were purified by dialysis using distilled water for 48 h, and then the unreacted small molecule compounds such as SD and RhB were enough to be eliminated. Experimental tests, such as IR, ^1^H NMR, ^13^C NMR, GPC, UV, and fluorescent assay, proved successful in the formation of dextran fluorescent probes (Figure 1, Figure 2 and Figure 3).

The typical peaks of RhB and the dextran group appeared in the ^1^H NMR and ^13^C NMR of Dextran-RhB and SD-Dextran-RhB. Meanwhile, the typical peaks of the SD group were also found in the ^1^H NMR and ^13^C NMR of SD-Dextran-RhB (Figure 1). The ^13^C NMR spectra of SD-Dextran-RhB indicated the typical peaks of the RhB groups to be at 152.99, 148.86, 130.66, 129.16, 128.66, 44.13, 26.60, and 12.90 ppm, the typical peaks of the dextran groups to be at 98.71 and 97.78 ppm, and the typical peaks of the SD groups to be at 157.74, 129.58, and 112.38 ppm (Figure 1b).

The grafted molar ratios of the SD and RhB groups in SD-Dextran-RhB were determined by the characteristic peaks of ^1^H NMR. The ^1^H NMR spectra of Dextran-RhB showed that the phenyl groups and methyl group of RhB emerged at 7.49–6.26 ppm and 1.08 ppm (Figure 1a), respectively, which elaborated that RhB was covalently bonded to dextran. The integral area ratio of 1.08 ppm (CH_3_ groups in RhB structure (12H)) to 3.21 ppm (CHCH_2_O groups in dextran repeat unit structures (2H)) was 0.12:1, so the grafting ratio of RhB structures in SD-Dextran-RhB can be calculated to be a 2.0% molar ratio to dextran repeat unit structures. The ^1^H NMR spectra showed that the pyrimidine ring of SD appeared at 8.14 ppm and that the phenyl groups of SD at 7.49–6.26 ppm were overlapped with the phenyl groups of RhB, indicating that the SD and RhB groups were covalently bonded to dextran (Figure 1b). The integral area ratio of 8.14 ppm (CH groups in SD structure (1H)) to 3.21 ppm (CHCH_2_O groups in dextran repeat unit structures (2H)) was 0.007:1, so the grafting ratio of SD can be determined to be a 1.4% molar ratio to dextran repeat unit structures.

The IR spectra of SD-Dextran-RhB showed the typical absorption peaks of carboxyl to be 1645–1527 cm^−1^ (Figure 2a), the hydroxyl groups of dextran to be 3440 cm^−1^, the phenyl groups to be RhB of 946–668 cm^−1^, and the pyrimidine ring groups of SD to be 1270 cm^−1^, indicating that both RhB and SD were covalently bonded to dextran. The differential scanning calorimeter (DSC) curves of SD-Dextran-RhB also changed and were different to those of the SD and RhB derivatives (Figure 2b). The decomposition temperatures of RhB and SD were 210–211 °C and 252–256 °C, respectively. The thermostability of SD-Dextran-RhB was reduced by the attachments of RhB and SD to dextran.

The molecular weight was measured using gel permeation chromatography (GPC). The first peak presented the normal average number weight of the sample. The other peaks appeared at the last period and eluted from GPC column later, which also presented the solvent of water. Average number molecular weight (Mn) of SD-Dextran-RhB was 5.51 × 10^3^ and the polydispersity index was 1.96 (Figure 3). The molecular aggregation structure of SD-Dextran-RhB in the solution changed due to the attachments of RhB and SD to dextran, which induced the reduction in Mn of SD-Dextran-RhB. Thus, these results exhibited that RhB and SD were covalently bonded to dextran.

### 3.2. Fluorescence and UV Spectra

**Figure 4:**
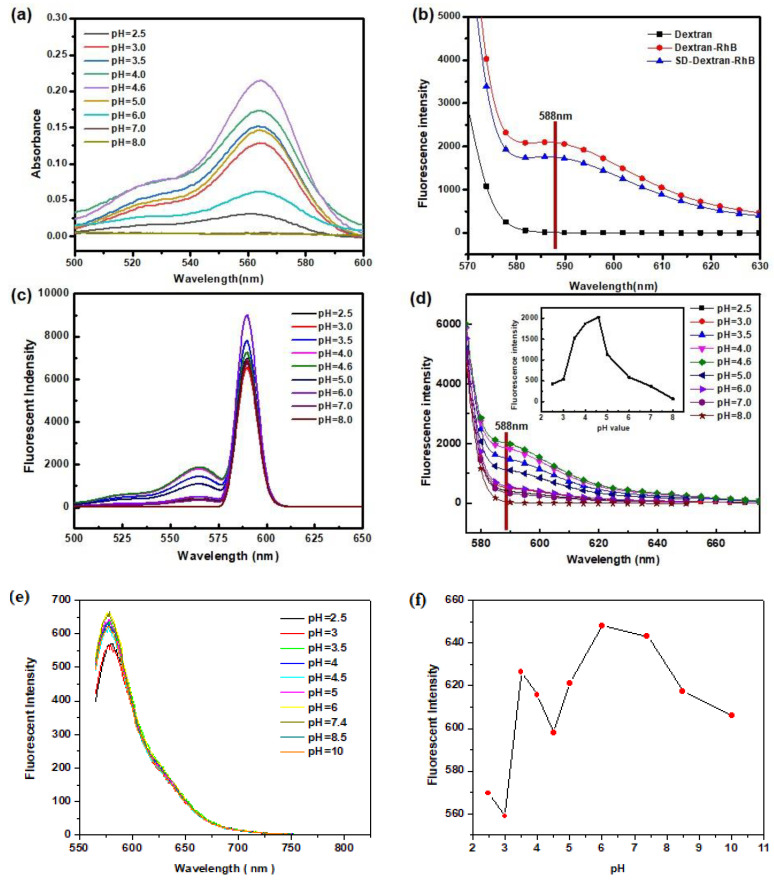
UV spectra (**a**), fluorescent spectra (**b**), fluorescent excitation spectra (**c**), and fluorescence emission spectra (**d**) of SD-Dextran-RhB and fluorescent excitation spectra (**e**,**f**) of RhB in different acidic conditions.

The UV and fluorescent spectra of SD-Dextran-RhB indicated the typical UV absorption peaks of SD and the fluorescent emission peaks of the RhB groups, respectively (Figure 4). The intensity of the UV absorption peak of SD-Dextran-RhB solutions (0.05 mg/mL) was enhanced at 565 nm, when their pH values increased from 2.5 to 4.6. However, the intensity of the UV absorption peak of the SD-Dextran-RhB solutions decreased, whilst their pH values continued increasing from 4.6 to 10. So, the highest UV intensity of the SD-Dextran-RhB solutions was reached when the pH value was 4.6.

The acquisition conditions of the fluorescent assay adopted the excitation slit width of 5 nm, emission slit width of 5 nm, and voltage of 600 V. The excitation wavelength is 546 nm, and the emission spectrum collection range is 566–800 nm. The sample test concentration is 1 × 10^−5^ mol/L. The relationship between the fluorescent emission intensity of RhB and pH value is shown in Figure 4e,f. With the increase in the pH value of the solution, the fluorescent intensity has an upward trend due to the balance of the anilinium cation and carboxyl groups in the RhB structure. The fluorescent intensity at 581 nm of RhB was weak when the pH value was 2–3, since the carboxyl group as a strong electron-withdrawing group was not ionized. When the pH value continued increasing, the carboxyl group began to ionize, and the electron absorption ability gradually decreased due to the gradual increase in ionization degree, which was induced in the enhanced fluorescent intensity.

The fluorescent emission peaks of Dextran-RhB and SD-Dextran-RhB were at 588 nm, using excitation with a maximum wavelength of 565 nm (Figure 4b). Moreover, SD-Dextran-RhB possessed sensitivity to the acidic conditions as well as the UV spectra. The fluorescent intensity of SD-Dextran-RhB solutions (0.05 mg/mL) at 588 nm increased when the pH values of the solutions increased from 2.5 to 4.6 and then decreased as the pH values increased from 4.6 to 8.0. The highest fluorescent intensity of the SD-Dextran-RhB solutions was reached when the pH value was 4.6. A curve of best fit was made according to the Henderson-Hasselbach equation, and the pKa of SD-Dextran-RhB was further calculated to be 4.92 (Figure 4d), which is related to the anilinium cation and intramolecular spirolactam of the RhB groups. Therefore, the UV and fluorescent spectra of SD-Dextran-RhB were affected by the pH values in the solutions.

The relationship between the fluorescence emission intensity and the pH value the in SD-Dextran-RhB solutions is shown as Figure 4c,d. When the pH value was 7.4–10, SD-Dextran-RhB displayed very weak fluorescent intensity at 588 nm. The fluorescent intensity gradually increased with the decrease in the pH value and reached the maximum value when the pH value was 4.6. This was probably due to the ring opening of the rhodamine ethylenediamine intramolecular spirolactam in the SD-Dextran-RhB structure, which was induced by hydrogen ions. When SD-Dextran-RhB interacted with the hydrogen ion, the UV absorption spectra and fluorescent spectra changed and realized the performance of the pH response, which is obviously different from pure RhB. These results are consistent with the reports cited in the literature [40,41].

An acidic environment is currently found in some tumors. However, normal cells possess a neutral environment. SD-Dextran-RhB displayed acidic sensitivity and high fluorescent property in an acidic environment. So, SD-Dextran-RhB can potentially be used as a fluorescent imaging probe to realize the enhanced imaging of tumors and differentiate normal cells. The work mechanism of acidic sensitivity was reported as pH mediated the ring opening of the intramolecular spirolactam of the RhB group, yielding a highly fluorescent species in an acidic environment, whilst the intramolecular spirolactam of the RhB group was displayed in neutral and basic environments [40,41].

### 3.3. Cell Cytotoxicity

**Figure 5:**
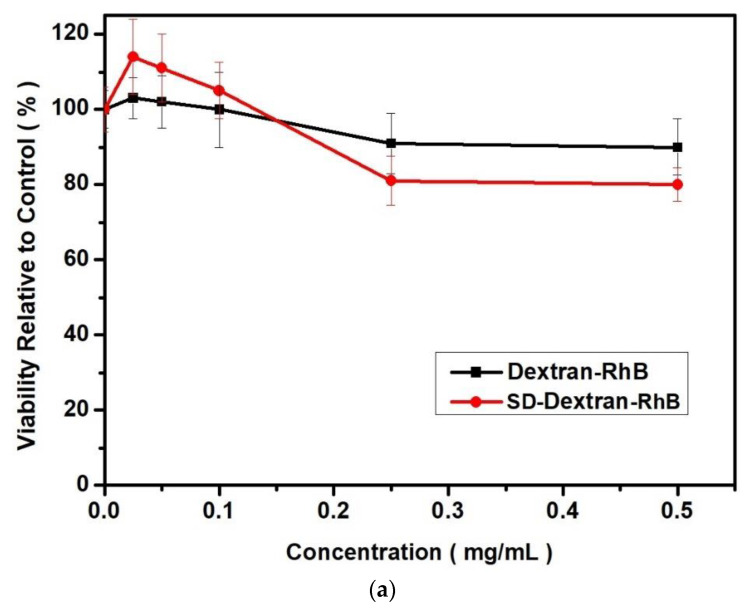

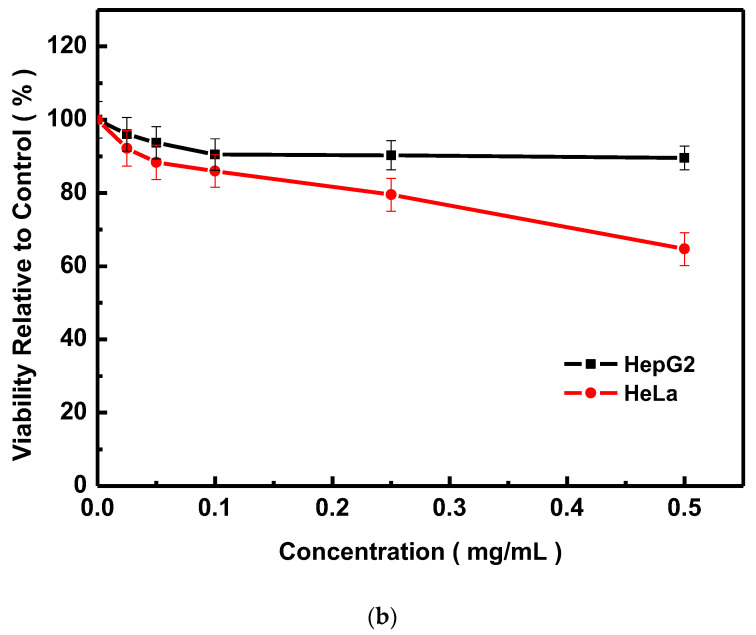
Cell toxicity assay of Dextran-RhB and SD-Dextran-RhB to normal 293 cells (**a**) and SD-Dextran-RhB (**b**) to HepG2 and HeLa cells.

The influence of SD-Dextran-RhB on normal 293, HepG2, and HeLa cell growth is listed in Figure 5. SD-Dextran-RhB and Dextran-RhB had low cytotoxicity to normal 293 cells. Meanwhile, SD-Dextran-RhB indicated lower cytotoxicity to normal 293 cells than Dextran-RhB, due to the introduction of the SD groups. The cytotoxicity to 293 cells incubated with Dextran-RhB and SD-Dextran-RhB were 90% and 80%, respectively, at 500 µg/mL of the same sample concentration in growth medium. HepG2 and HeLa cells were incubated with different concentrations of SD-Dextran-RhB in growth medium. SD-Dextran-RhB demonstrated low cytotoxicity to HeLa and HepG2 cells. Moreover, the cytotoxicity to HeLa and HepG2 cells became higher when the concentration of SD-Dextran-RhB increased. Cell viability of HepG2 and HeLa cells at 500 µg/mL of SD-Dextran-RhB concentration was 89.6% and 64.7%, respectively, relative to the control. Meanwhile, SD-Dextran-RhB possessed lower cytotoxicity to HepG2 cells than to HeLa cells, after 48 h incubation at the same concentration. Therefore, SD-Dextran-RhB displayed lower cytotoxicity to normal 293 cells than to HeLa tumors cells.

### 3.4. Cell Uptake Assay

**Figure 6:**
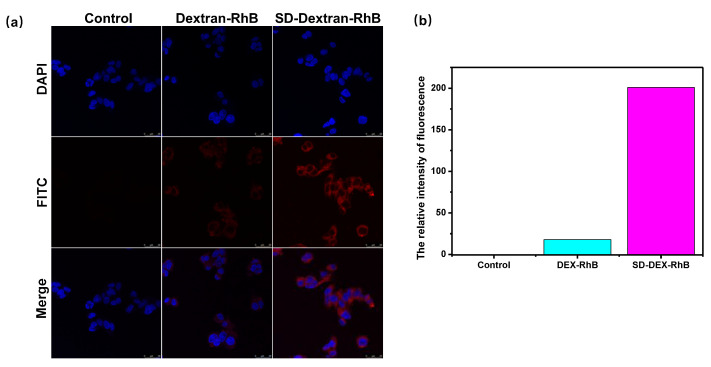
Confocal microscopy images (**a**) and relative intensity of fluorescent intensity (**b**) in HepG2 cells cultured with growth medium, Dextran-RhB, and SD-Dextran-RhB for 2 h.

HepG2 cells incubated with SD-Dextran-RhB, Dextran-RhB, and pure growth medium were observed using a fluorescence confocal microscope. Cell morphologies under fluorescent imaging, when excited by DAPI channel (405 nm) and FITC channel (561 nm), are listed in Figure 6. HepG2 cells incubated with SD-Dextran-RhB displayed a noticeably cleaner red fluorescent imaging than Dextran-RhB. Meanwhile, the fluorescent intensity of HepG2 cells cultured with SD-Dextran-RhB was obviously higher than that of HepG2 cells incubated with SD-Dextran and pure growth medium. Dextran-RhB was taken up by HepG2 cells through the tumor EPR effect and galactoside molecules on the surface, since dextran is one of the polysaccharide carriers and Dextran-RhB displayed the micelles in aqueous solution. So, red fluorescent imaging started to be visible in the FITC condition for Dextran-RhB. SD-Dextran-RhB indicated a high specific uptake by tumor cells, due to the receptor-mediated action of the SD groups. Through this special action, SD-Dextran-RhB molecules can be introduced into tumor cells. Thus, this result demonstrated that SD-Dextran-RhB possessed desirable a fluorescent imaging property for HepG2 cells. Moreover, SD-Dextran-RhB demonstrated a high selective uptake by HepG2 cells, due to the tumor-targeting property of the SD group.

### 3.5. Fluorescent Imaging

**Figure 7:**
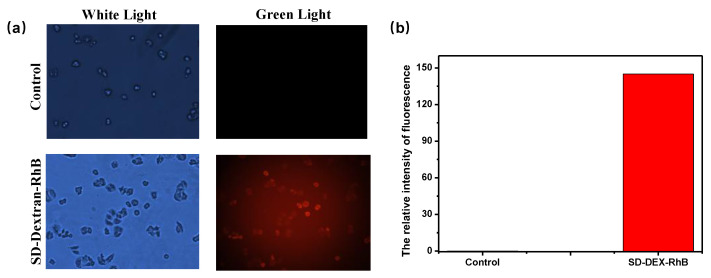
Fluorescent imaging (**a**) and relative fluorescent intensity (**b**) of SD-Dextran-RhB in HeLa cells.

Fluorescent imaging of HeLa cells cultured with SD-Dextran-RhB and growth medium were observed using a fluorescent inverted microscope. Their cell morphologies under fluorescent imaging, when excited by white and green light (excitation wavelength: 565 nm), are listed in Figure 7, respectively. HeLa cells cultured with growth medium in the control indicated a good growth condition, as seen when excited by white light, but no fluorescent image could be observed when excited by green light. On the other hand, HeLa cells cultured with SD-Dextran-RhB indicated an obviously good red fluorescent image when excited by green light. Thus, this result demonstrated that SD-Dextran-RhB possessed a desirable fluorescence property in the imaging of HeLa cells. Meanwhile, SD-Dextran-RhB displayed a high selective uptake by HeLa cells, due to the tumor-targeting property of the SD group.

## 4. Conclusions

Dextran fluorescent compounds (SD-Dextran-RhB) were prepared by the incorporation of RhB and SD to dextran as a polymer carrier. SD-Dextran-RhB demonstrated sensitivity to acidic conditions and low cytotoxicity to normal 293 cells and HepG2 and HeLa tumor cells. Moreover, SD-Dextran-RhB had good fluorescent imaging for HepG2 and HeLa tumors.

Currently some fluorescent compounds with low molecular weight, such as RhB and ICG, have been used to improve the imaging contrast and sensitivity in fluorescent imaging; however, since these probes are excreted from the body quickly, they then possess significant restrictions for imaging. Therefore, SD-Dextran-RhB can be used as a potential probe for fluorescent imaging.

## Data Availability

Not applicable.

## References

[1] Anderson C.J., Lewis J.S. (2017). Current status and future challenges for molecular imaging. Phil. Trans. R. Soc. A.

[2] Deng Y., Xu A., Yu Y., Fu C., Liang G. (2019). Biomedical applications of fluorescent and magnetic resonance imaging dual-modality probes. ChemBioChem.

[3] Zhang Y.B., Sun L., Yan Q., Qiu X.Y., Cheng Y.T., Wang B.L., Tan X.P., Fang M.X., Luck R.L., Liu H.Y. (2022). Near-infrared fluorescent probe based on cyanine scaffold for sensitive detection of uranyl ions in living cells and water samples. Microchem. J..

[4] He S., Song J., Qu J., Cheng Z. (2018). Crucial breakthrough of second near-infrared biological window fluorophores: Design and synthesis toward multimodal imaging and theranostics. Chem. Soc. Rev..

[5] Huang X., Song J., Yung B.C., Huang X., Xiong Y., Chen X. (2018). Ratiometric optical nanoprobes e;nable accurate molecular detection and imaging. Chem. Soc. Rev..

[6] Su D., Teoh C.L., Wang L., Liu X., Chang Y.T. (2017). Motion-induced change in emission (MICE) for developing fluorescent probes. Chem. Soc. Rev..

[7] Zhao H., Zhao H., Jiao Y., Zhu Y., Liu C., Li F., Wang Y., Gu Z., Yang D. (2020). Biosynthetic molecular imaging probe for tumor-targeted dual-modal fluorescence/magnetic resonance imaging. Biomaterials.

[8] Zhang Z., Hong H., Tao T., Jie L., Li Y. (2018). Rational design of nanoparticles with deep tumor penetration for effective treatment of tumor metastasis. Adv. Funct. Mater..

[9] Liang S.C., Liu Y.M., Fu T., Yang F., Chen X.H., Yan G.P. (2016). A water-soluble and biocompatible polymeric nanolabel based on naphthalimide grafted poly(acrylic acid) for the two-photonfluorescence imaging of living cells and C. elegans. Colloids Sur. B Biointerfaces.

[10] Liang S.C., Tong Q., Qin X.N., Liao X.Y., Li Q., Yan G.P. (2020). A hydrophilic naphthalimide-based fluorescence chemosensor for Cu^2+^ ion: Sensing properties, cell imaging and molecular logic behavior. Spectrochim. Acta Part A Mol. Biomol. Spectrosc..

[11] Li H., Parigi G., Luchinat C., Meade T.J. (2019). Bimodal fluorescence-magnetic resonance contrast agent for apoptosis imaging. J. Am. Chem. Soc..

[12] Liao X.Y., Liang S.C., Liu Y.P., Zhang J.Y., Yan G.P. (2017). Preparation, characteristics and cell imaging of fluorescent nanoparticles based on grafted poly(acrylic acid). Chin. J. Anal. Chem..

[13] Zhao J., Jin G., Weng G., Li J., Zhu J., Zhao J. (2017). Recent advances in activatable fluorescence imaging probes for tumor imaging. Drug Discov. Today.

[14] Wang C., Wang Z., Zhao T., Li Y., Huang G., Sumer B.D., Gao J. (2018). Optical molecular imaging for tumor detection and image-guided surgery. Biomaterials.

[15] Wang W., Hu Z. (2019). Targeting peptide-based probes for molecular imaging and diagnosis. Adv. Mater..

[16] Liu Z., Wang H., Sun C., He Y., Xia T., Wang J., Xiong X., Zhang Q., Yang S., Liu L. (2022). ZWZ-3, a fluorescent probe targeting mitochondria for melanoma imaging and therapy. Front. Pharmacol..

[17] Liu F., Shen Y.C., Chen S., Zhang Q., Guo Q.Z., Yan G.P., Zhang Q., Guo Q.Z., Gu Y.T. (2020). Tumor-targeting fluorescent probe based on 1,8-naphthalimide and porphyrin groups. Chemistryselect.

[18] Zheng S.Y., Tang W.Q., Zhang M., Yan J.R., Liu F., Yan G.P., Liang S.C., Wang Y.F. (2022). Dual-modal polypeptide-containing contrast agents for magnetic resonance/fluorescence imaging. Bioorganic Chem..

[19] Liu F., Yan J.R., Chen S., Yan G.P., Pan B.Q., Zhang Q., Wang Y.F., Gu Y.T. (2020). Polypeptide-rhodamine B probes containing laminin/fibronectin receptor-targeting sequence (YIGSR/RGD) for fluorescent imaging in cancers. Talanta.

[20] Ouyang Y.H., Xu W., Zhou C.K., Liu F., Li B., Yan G.P., Yang L., Chen S., Jiang C. (2018). Porphyrin-containing gadolinium complex as a tumor-targeting magnetic resonance imaging (MRI) contrast agent. Curr. Drug Deliv..

[21] Liu F., Zou T.J., Tan Z.L., Chen S., Wu Z.H., Yan G.P., Zhang Q., Liang S.C., Yang J. (2017). Isoindoline nitroxide-labeled porphyrins as potential fluorescence-suppressed spin probes. Org. Biomol. Chem..

[22] Liu F., Zou T.J., Tan Z.L., Yan G.P., Guo J.F., Zhang Q., Liu H., Yang J. (2016). Fullerenol spin probe containing isoindoline nitroxide and porphyrin groups. Fuller. Nanotub. Car. N.

[23] Liu F., Shen Y.C., Ouyang Y.H., Yan G.P., Chen S., Liu H., Wu Y.G., Wu J.Y. (2017). Synthesis and properties of isoindoline nitroxides-containing porphyrins. J. Heterocycl. Chem..

[24] Wang J., Dong R., Wu H., Cai Y., Ren B. (2020). A review on artificial micro/nanomotors for cancer-targeted delivery, diagnosis, and therapy. Nano-Micro Lett..

[25] Jin S.Y., Lin L.P., Chen X.H., Liu F., Zhu X.B., Yan G.P. (2021). Preparation and properties of fluorescent quantum dots microbeads encapsulated in-situ by polyisobornyl methacrylate for immunoch romatography. J. Nanophoton..

[26] Yu X.Q., Yu K.K., Li K., Lu C.Y., Bao J. (2020). Multifunctional gold nanoparticles as smart nanovehicles with enhanced tumour-targeting abilities for intracellular pH mapping and in vivo MR/fluorescence imaging. Nanoscale.

[27] Gwang J.N., Hongsuk P., Eun S.L. (2018). Augmented tumor accumulation and photothermal ablation using gold nanoparticles with a particular cellular entry orientation. J. Bioact. Compat. Polym..

[28] Zhang M., Liu F., Ke X.J., Chen S., Yan G.P., Zhang Q., Liang S.C., Wang Y.F., Jiang C. (2020). Polyaspartamide fluorescent probe containing rhodamine B and Sulfadiazine groups. Chin. J. Org. Chem..

[29] Zhu Y.L., Shen Y.C., Liu F., Chen S., Yan G.P., Liang S.C., Zhang Y.F., Wu Y.G. (2021). Dual-modal fullerenol probe containing glypican-3 monoclonal antibody for electron paramagnetic resonance/fluorescence imaging. Fuller. Nanotub. Car. N.

[30] Zhang Y., García-Gabilondo M., Grayston A., Feiner I.V., Anton-Sales I., Loiola R.A., Llop J., Ramos-Cabrer P., Barba I., Garcia-Dorado D. (2020). PLGA protein nanocarriers with tailor-made fluorescence/MRI/PET imaging modalities. Nanoscale.

[31] Du H.J., Shen Y.C., Liu Y.P., Han L., Zheng Y., Yan G.P., Tu Y.Y., Wu J.Y., Guo Q.Z., Zhang Y.F. (2015). Dextran gadolinium complex containing folate groups as a potential magnetic resonance imaging contrast agent. Chin. J. Polym. Sci..

[32] Yan G.P., Xu W., Yang L., Li L., Liu F., Guo Q.Z. (2010). Dextran gadolinium complexes as contrast agents for magnetic resonance imaging to sentinel lymph nodes. Pharm. Res..

[33] Li Q., Han L., Yan G.P., Tu Y.Y., Yuan H., Zou T.J., Shao C.T., Liu H.F. (2014). Studies on dextran gadolinium complex containing Sulfadiazine groups. Acta. Polym. Sin..

[34] Huang J.L., Wang H.Y., Zhou C.L. (1995). Ring-opening polymerization of ethylene oxide by anion initiation using sulfadiazine as parents compound. J. Appl. Polym. Sci..

[35] Liu F., Zhao B., Xia X.T., Yan J.R., Yu F.Q., Yan G.P., Hu J., Chen S., Wang Y.F., Liu H. (2019). Al^18^F labeled sulfonamide-conjugated PET tracer in vivo tumor-targeted imaging. J. Cell Biochem..

[36] Yan G.P., Zong R.F., Li L., Fu T., Liu F., Yu X.H. (2010). Nano anticancer drug loaded nanospheres based on biodegradable amphiphilic ε-caprolactone and carbonate copolymers. Pharm. Res..

[37] Pal R.R., Kim M.S., Lee D.S. (2005). Synthesis and pH-dependent micellization of sulfonamide-modified diblock copolymer. Macromol. Res..

[38] Yan G.P., Liu M.L., Li L.Y. (2005). Studies on polyaspartamide gadolinium complexes containing sulfadiazine groups as MRI contrast agents. Bioconjugate Chem..

[39] Shi F.Q. (1990). How to copy the disease model of animals. Medical Animal Experiment Method.

[40] Urano Y., Asanuma D., Hama Y., Koyama Y., Barrett T., Kamiya M., Nagano T., Watanabe T., Hasegawa A., Choyke P.L. (2009). Selective molecular imaging of viable cancer cells with pH-activatable fluorescence probes. Nat. Med..

[41] Kim H.N., Lee M.H., Kim H.J., Kim J.S., Yoon J.Y. (2008). A new trend in rhodamine-based chemosensors: Application of spirolactarn ring-opening to sensing ions. Chem. Soc. Rev..

